# Sleep Apnea Combined with Pulmonary Hypertension in a Veteran Patient Population

**DOI:** 10.3390/jcm12144634

**Published:** 2023-07-12

**Authors:** Paul Stark, Eric Y. Chang

**Affiliations:** 1 Radiology Service, VA San Diego Healthcare System, San Diego, CA 92161, USA; ericchangmd@gmail.com; 2 Department of Radiology, University of California, San Diego, CA 92093, USA

**Keywords:** sleep apnea, pulmonary hypertension, CT scanning

## Abstract

We have investigated the concurrence of sleep apnea and pulmonary hypertension in a Veteran population. We retrospectively reviewed 142 patients who underwent chest CT scans and had a dilated main pulmonary artery, defined as a width exceeding 29 mm on axial images. Approximately 40% of patients with pulmonary hypertension had associated sleep apnea. No significant difference in pulmonary artery diameters could be found between the group without sleep apnea and the group with sleep apnea (34.5 ± 4.2 mm vs. 34.7 ± 4.4 mm, *p* = 0.373).

## 1. Introduction

Sleep apnea and pulmonary hypertension are two conditions that occur with high frequency in an elderly population [1]. Sleep apnea is well recognized as a cause of hypoxia and hypercarbia with subsequent hypoxic pulmonary vasoconstriction, particularly once the alveolar partial pressure for oxygen drops below a threshold of 60 mm Hg. This is known as the von Euler–Liljestrand reflex, whereby pulmonary vascular resistance is increased in regions that are hypoventilated in an attempt to avoid ventilation–perfusion mismatch [2].

This useful, physiologic response can become pathologic in sleep apnea and lead to chronic pulmonary hypertension and cor pulmonale [3,4]. We have observed a dilated main pulmonary artery exceeding 29 mm in width on routine chest CT scans performed for diverse indications but segregated those patients with sleep apnea as a potential explanation for the pulmonary hypertension.

## 2. Materials and Methods

This study has Institutional Review Board approval. We retrospectively reviewed 142 consecutive axial thoracic CT scans in patients with a dilated main pulmonary artery measuring at least 29 mm in transverse diameter or width. The CT criteria for the diagnosis of pulmonary hypertension hinge on identification of an enlarged main pulmonary artery with a width of more than 29 mm as measured in the axial plane, proximal to its bifurcation and orthogonal to the long axis of the main pulmonary artery. Widths of 29–31 mm indicate mild pulmonary hypertension, 31–34 mm indicate moderate pulmonary hypertension and larger than 34 mm Hg indicate severe pulmonary hypertension [5]. The width of a dilated pulmonary artery can exceed the diameter of the ascending aorta at the same level. Dilation of the right ventricle, right atrium, coronary sinus and tricuspid regurgitation are further indicators of pulmonary hypertension on CT scans. Overall, CT has an acceptable performance for the diagnosis of pulmonary hypertension with a sensitivity of 87% and a specificity of 89% [6]. In comparison, transthoracic echocardiography has a sensitivity of 85% and specificity of 74% and transesophageal echocardiography has a sensitivity of 84% and specificity of 83% [6]. In addition, we reviewed the electronic health records (CPRS) for the presence or absence of the diagnosis of sleep apnea.

The Kolmogorov–Smirnov test was used to test for normality and the appropriate test was used to determine if differences existed between of the two groups. A *p*-value less than 0.05 was considered statistically significant. Analyses were performed using SPSS (Version 21.0 IBM SPSS, Chicago, IL, USA).

## 3. Results

**Figure 1:**
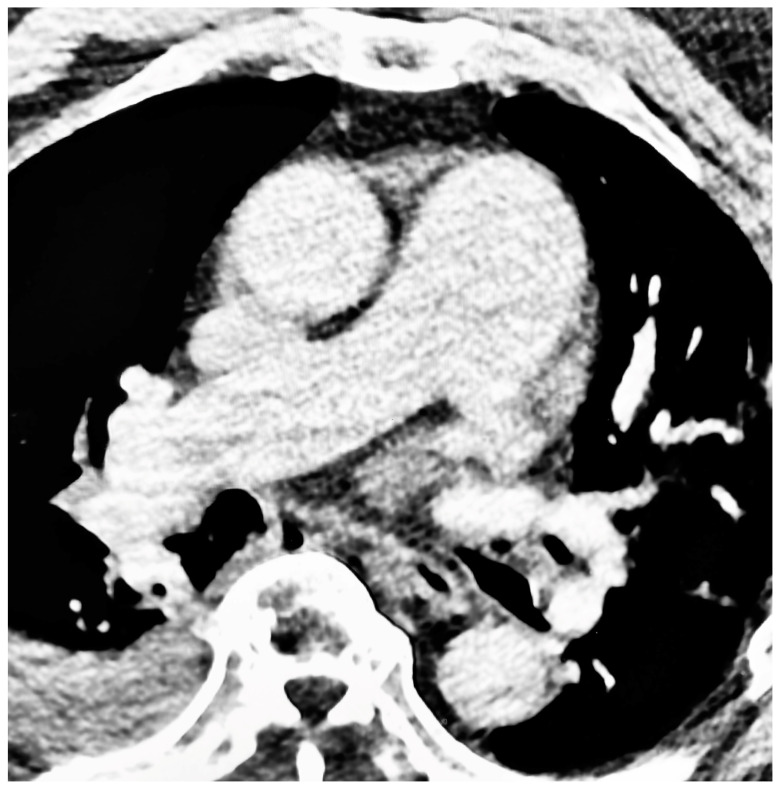
Pulmonary hypertension in a patient with sleep apnea. Axial, contrast-enhanced CT scan of the thorax with a section taken at the level of the main pulmonary artery bifurcation. The main pulmonary artery has a width of 49 mm. Incidental note is made of a moderate right pleural effusion.

A total of 142 patients were identified with a dilated main pulmonary artery on axial images. Of these, 57 patients, or 40%, had a history of sleep apnea (Figure 1). The vast majority of our diagnosed sleep apnea patients had obstructive sleep apnea.

Patients with a dilated main pulmonary artery had similar characteristics independent of the presence or absence of sleep apnea. The mean age of our patients with sleep apnea was 67.9+/−9.9 years. The mean age of patients with a dilated pulmonary artery but without a history of sleep apnea was 67.7+/−12.9 years. The mean width of the main pulmonary artery in patients with sleep apnea was 34.7+/−4.4 mm. The mean width of the pulmonary artery in patients without sleep apnea was 34.5+/−4.2 mm.

**Figure 2:**
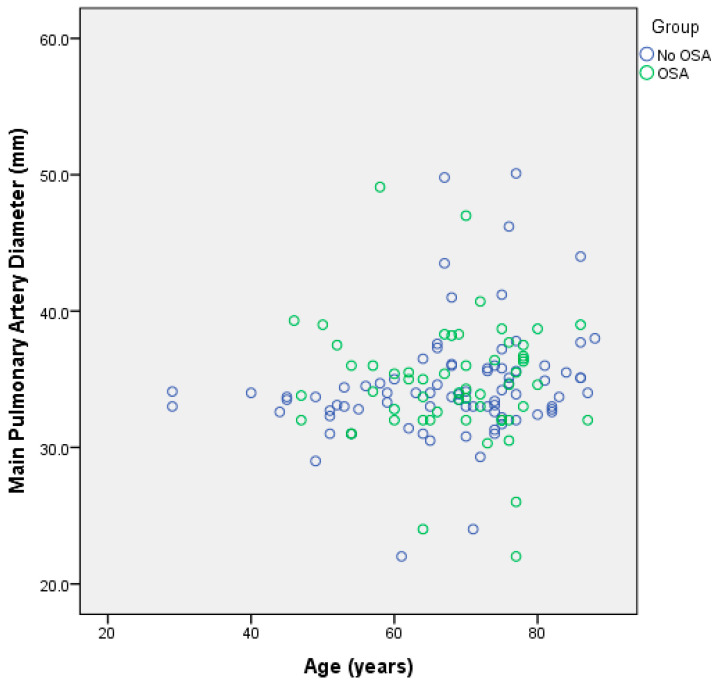
Scatter plot showing that there is no substantial difference in pulmonary artery diameter or age between the group without sleep apnea or the group with sleep apnea.

**Figure 3:**
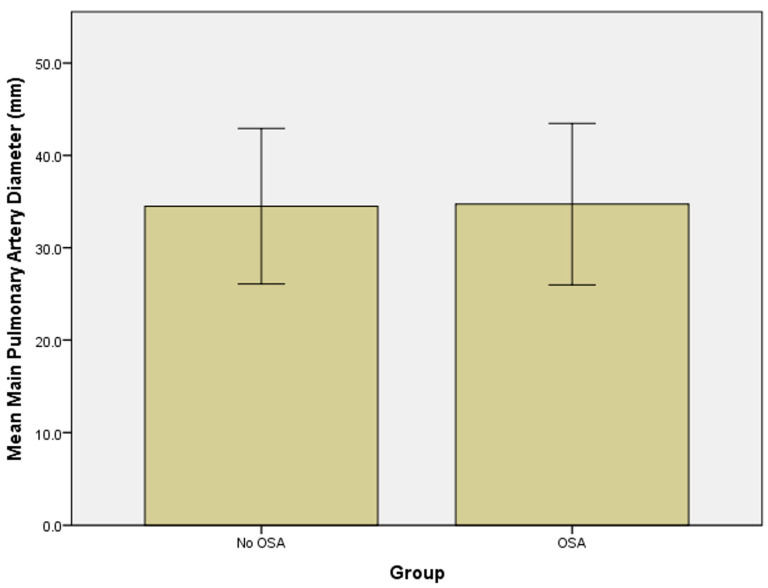
Bar graphs showing the mean pulmonary artery diameter with 2 standard deviation error bars in the group without sleep apnea and the group with sleep apnea.

Using the Mann–Whitney test, no significant differences in age between the two groups were identified (*p* = 0.872) (Figure 2). No significant difference in main pulmonary artery width between the two groups of patients was found (*p* = 0.373) (Figure 3).

## 4. Discussion

Pulmonary hypertension is defined as increased pressure in the pulmonary circulation with a systolic pressure at rest of 18–25 mm Hg, diastolic pressure of 10 mm Hg and mean pulmonary artery pressure of 12–16 mm Hg at sea level, rising to 30/13 mm Hg (mean 20 mm Hg) with mild exercise. Pulmonary artery pressures rise with altitude: at 15,000 feet, normal resting pulmonary artery pressures are about 38/14 mm Hg [2,4,7].

Pulmonary hypertension is classified by the World Health Organization into five categories, according to etiology, pathophysiology or histopathology, clinical characteristics and hemodynamic profile [2,3,4]. Group 1 pulmonary hypertension, also called idiopathic pulmonary hypertension, is characterized by plexiform or plexogenic lesions as a result of angioproliferative activity of endothelial cells in tandem with hyperplasia and hypertrophy of the smooth muscles in precapillary arterioles. Pulmonary arterial hypertension requires that the left ventricular filling pressure, pulmonary capillary wedge pressure, left atrial pressure and left ventricular end-diastolic pressure be 15 mm Hg or less, and that the calculated pulmonary vascular resistance be greater than 3 Woods Units (WU), which is defined as pulmonary arterial pressure minus capillary wedge pressure divided by the cardiac output (e.g., 25 − 10/5 = 3 WU). Group 2 pulmonary hypertension is due to left heart disease with increased back pressure, elevated diastolic left ventricular pressure, elevated pulmonary capillary wedge pressure and passive post-capillary pulmonary hypertension. This is usually the result of left ventricular failure with preserved or reduced ejection fraction or mitral valve disease. Group 3 pulmonary hypertension is due to lung parenchymal disease or hypoxia caused by sleep apnea, disordered breathing, high altitude or severe scoliosis. Hypoxic pulmonary vasoconstriction occurs once the alveolar partial pressure for oxygen reaches 60 mm Hg or less (von Euler–Liljestrand reflex). Group 4 includes chronic thrombo-embolic pulmonary hypertension (CTEPH) as a sequela of acute pulmonary embolism. This complication happens in 3.8% of patients who survive an episode of acute pulmonary embolism. CTEPH is characterized by bronchial collateralization. Group 5 pulmonary hypertension is due to multifactorial mechanisms including lymphangioleiomyomatosis, sarcoidosis, pulmonary Langerhans cell histiocytosis and fibrosing mediastinitis, among other etiologies.

Sleep apnea can be central in origin in 5% of affected patients but 95% have obstructive sleep apnea [8]. Both variants can lead to pulmonary hypertension via the hypoxic vasoconstriction mechanism detailed in Group 3 of the WHO classification. In the literature, between 20% and 30% of patients with obstructive sleep apnea develop pulmonary hypertension [8,9,10]. In addition, 10–15% of patients with obstructive sleep apnea have a concurrent diagnosis of chronic obstructive pulmonary disease [8]. Of note, a high percentage of patients with idiopathic pulmonary fibrosis have accompanying sleep apnea (62%) [10].

Obstructive sleep apnea occurs in all age groups but with increasing prevalence in people over the age of 60 years, mostly driven by obesity. At the current time, 42.4% of the US population is obese with a body mass index (BMI) exceeding 30 [8,11].

Obstructive sleep apnea is the most common sleep-related respiratory disorder [12]. Increased upper airway compliance is due to intermittent relaxation of the tongue, soft palate and pharyngeal muscles with resultant increased upper airway resistance and subsequent complete blockage of the upper airways. A large tongue, redundant soft palate tissue and large tonsils can combine to form a “crowded oropharynx”. Restricted chest wall movements can lead, in 10–20% of cases, to severe alveolar hypoventilation with obesity hypoventilation syndrome (OHS) [13], which in turn leads to intermittent elevation of the arterial partial pressure of CO_2_ exceeding 55 mm Hg and concurrent hypoxemia. Each obstructive episode leads to negative intrathoracic pressure, increased afterload of both right and left ventricles, decreased left ventricular compliance, increased pulmonary artery pressure, decreased coronary artery blood flow and increased myocardial oxygen demand [8].

Forty million Americans have obstructive sleep apnea, including 3–7% of men and 2–5% of women [3]. Pulmonary hypertension is present in 12–34% of obstructive sleep apnea [14]. Conversely, 25% of patients with pulmonary hypertension by right heart catheterization turn out to have sleep apnea [12]. In our group of patients, the percentage of patients with sleep apnea and with associated pulmonary hypertension based on radiologic criteria, was higher than reported in the literature [7,10] and reached 40% of patients with dilated main pulmonary arteries.

Obesity is prevalent in Veterans. Chronic obstructive pulmonary disease, long-term exposure to chemicals and dust during military service, as well as post-traumatic stress disorder, favor the development of obstructive sleep apnea [15]. These patients are at a 79% higher risk of developing cardiovascular disease [15,16].

In conclusion, we demonstrate a higher prevalence of sleep apnea in U.S. Veterans with pulmonary hypertension as detected on CT scans than previously reported. No significant difference in pulmonary artery diameters could be found between the group without sleep apnea and the group with sleep apnea (34.5 ± 4.2 mm vs. 34.7 ± 4.4 mm, *p* = 0.373).

## Data Availability

Data is available upon written request.
